# Supplementary material for: Impaired sensory evidence accumulation and network function in Lewy body dementia
Source: Brain Commun. 2021 Aug 2;3(3):fcab089. doi: 10.1093/braincomms/fcab089 (PMC8361397; doi:10.1093/braincomms/fcab089)
Supplement: fcab089_Supplementary_Data [file fcab089_supplementary_data.zip › Supplementary_material.docx]

**Supplementary Material**

**Impaired sensory evidence accumulation and network function in Lewy body dementia**

O’Callaghan et al.

Demographics and clinical characteristics for the Parkinson’s disease

dementia and dementia with Lewy bodies cases Page 2

*Supplementary Table 1.*

Comparison of DVARS and motion parameters across groups Page 4

*Supplementary Table 2.*

Comparison of number of no response trials across groups Page 5

*Supplementary Fig 1.*

Plot of posterior predictive against observed data Page 6

*Supplementary Fig. 2*

**Demographics and clinical characteristics for the Parkinson’s disease dementia and dementia with Lewy bodies cases**

**Supplementary Table 1.** Mean (standard deviation) for demographics and clinical characteristics.

| **Demographics &**  **clinical characteristics** | **Controls** | **AD** | **DLB** | **PDD** | | **Group**  ***p* value** | | **Post hoc**  ***p* value** | |  |
| --- | --- | --- | --- | --- | --- | --- | --- | --- | --- | --- |
| **N** | 23 | 16 | 18 | | 13 | | - | | - | |
| **Sex (M:F)** | 16:7 | 13:3 | 12:6 | | 13:0 | | - | | - | |
| **Age**  **Education** | 76.3 (5.4)  11.4 (1.8) | 76.4 (8.2)  10.6 (1.4) | 77.2 (5.5)  10.1 (1.6) | | 74.3 (4.4)  10.9 (1.3) | | n.s.  n.s. | | -  - | |
| **MMSE** | 29.1 (0.8) | 22.7 (3.0) | 23.9 (3.9) | | 24.2 (2.9) | | *** | | AD vs. Con^***^  DLB vs. Con^***^  PPD vs. Con^***^  DLB vs. AD^n.s.^  PDD vs. AD^n.s.^  DLB vs. PDD^n.s.^ | |
| **CAMCOG** | 96.8 (3.5) | 71.6 (12.2) | 76.5 (15.0) | | 79.5 (6.1) | | *** | | AD vs. Con^***^  DLB vs. Con^***^  PPD vs. Con^***^  DLB vs. AD^n.s.^  PDD vs. AD^*^  DLB vs. PDD^n.s.^ | |
| **UPDRS-III** | 1.3 (1.7) | 2.0 (1.6) | 14.3 (4.7) | | 25.6 (6.3) | |  | | AD vs. Con^n.s.^  DLB vs. Con^***^  PPD vs. Con^***^  DLB vs. AD^***^  PDD vs. AD^***^  DLB vs. PDD^***^ | |
| **CAF total** | - | 0.3 (0.8) | 3.3 (3.9) | | 5.8 (3.6) | | *** | | DLB vs. AD^*^  PDD vs. AD^***^  DLB vs. PDD^n.s.^ | |
| **Mayo total** | - | 9.0 (4.6) | 11.71 (6.3) | | 15.5 (5.7) | | * | | DLB vs. AD^n.s.^  PDD vs. AD^*^  DLB vs. PDD^n.s.^ | |
| **Mayo cognitive** | - | 2.1 (1.9) | 2.1 (1.8) | | 3.5 (2.0) | | n.s. | |  | |
| **Mayo arousal** | - | 0.9 (1.0) | 1.9 (1.5) | | 2.9 (1.0) | | *** | | DLB vs. AD^n.s.^  PDD vs. AD^***^  DLB vs. PDD^n.s.^ | |
| **Cholinest. Inhibitors** | - | 16(100%) | 16(89%) | | 11(85%) | | - | | - | |
| **Dopaminergic meds.** |  | - | 8 (42%) | | 13 (100%) | | - | | - | |
| **LEDD (mg/day)** | - | - | 350 (258) | | 889 (423) | | - | | ** | |

n.s. = non significant; *** = p < .001; ** = p < .01; * = p < .05.

Significance tests refer to between group one-way ANOVAs and post hoc Sidak-corrected pairwise t-tests. AD = Alzheimer’s disease; DLB = Dementia with Lewy body; PDD = Parkinson’s disease dementia; MMSE = Mini-Mental State Examination; CAMCOG = Cambridge Cognitive Examination; UPDRS-III = Unified Parkinson’s disease rating scale motor component; CAF total = Clinician Assessment of Fluctuation total score; Mayo total = Mayo Fluctuations Scale; Mayo cognitive = Mayo Fluctuation cognitive subscale; Mayo arousal = Mayo Fluctuations arousal subscale; LEDD = levodopa equivalent daily dose.

**Comparison of DVARS and motion parameters**

**Supplementary Table 2.** Mean (standard deviation) for DVARS and motion parameters.

|  | **Controls** | **AD** | **LBD** | ***F***  **value** | | ***p***  **value** |
| --- | --- | --- | --- | --- | --- | --- |
| **Mean xyz motion (mm)** | 0.14 (0.04) | 0.15 (0.06) | 0.18 (0.11) | | 0.89 | 0.42 |
| **Max xyz motion (mm)** | 0.55 (0.22) | 0.82 (0.41) | 0.71 (0.47) | | 1.73 | 0.19 |
| **Mean angular motion (°)**  **Max angular motion (°)** | 0.06 (0.01)  0.46 (0.29) | 0.11 (0.07)  0.80 (0.59) | 0.09 (0.08)  0.59 (0.61) | | 2.51  1.57 | 0.09  0.22 |
| **Mean DVARS** | 1.23 (0.11) | 1.26 (0.11) | 1.23 (0.087) | | 0.65 | 0.52 |

Mean (standard deviation); Significance tests refer to between group one-way ANOVAs

AD = Alzheimer’s disease; LBD = Lewy body disease.

**Comparison of number of no response trials across groups**

The amount of no response trials excluded from the analysis did not differ significantly across the groups [F(2, 35) = 1.97, *p* = 0.154].

**Supplementary Figure 1.**

**Plot of posterior predictive against observed data**

Posterior predictive checks were conducted by averaging 500 simulations generated from the model’s posterior to confirm it could reliably reproduce patterns in the observed data (Wiecki *et al.*, 2013).

**Supplementary Figure 2.**

Histogram shows observed reaction time distribution (positive vs. negative results on the x-axis correspond to left vs. right responses). The black line plots the results from the posterior predictive simulation, i.e., data simulated based on using 500 parameter values from the posterior to simulate a different data set for each parameter value.

**References**

Wiecki TV, Sofer I, Frank MJ. HDDM: Hierarchical Bayesian estimation of the Drift-Diffusion Model in Python. Front Neuroinform 2013; 7: 14.
