# Supplementary material for: Impaired sensory evidence accumulation and network function in Lewy body dementia
Source: Brain Commun. 2021 Aug 2;3(3):fcab089. doi: 10.1093/braincomms/fcab089 (PMC8361397; doi:10.1093/braincomms/fcab089)
Supplement: fcab089_Supplementary_Data [file Original Submission-Authors_Response.pdf › Original Submission-Authors_Response.pdf]

Reviewer: 1

Comments to the Author:

Interesting and competent work, well presented.

Typos:
Imaging acquisition -> Image acquisition?

We thank the Reviewer for their positive appraisal of the manuscript. We have corrected this typo.

Reviewer: 2

Comments to the Author:

The authors use a modified attentional task and computational modelling to assess sensory evidence accumulation in patients with Lewy body disease compared to Alzheimer's patients and controls. The attentional task and drift diffusion modelling are well described and well designed to test the authors hypothesis. The authors find impaired evidence accumulation in LBD but no correlation between drift rates and measures of fluctuation or cognitive measures. The findings are interesting and the additional correlation with resting state functional networks provides further useful insights into the cognitive mechanisms of LBD. However, there are few areas that further methodological information is needed.

We appreciate the Reviewer's positive comments on the manuscript and thank them for identifying areas where methodological details and Discussion points were lacking. We have addressed these as described in the points below.

Major comments:

1. The authors rightly include both patients with Parkinson's disease dementia and Dementia with Lewy bodies in their LBD patient group; although underlying pathophysiology is likely to be similar in both groups they may differ in key factors, particularly in the degree of cognitive impairment, the presence and severity of fluctuations and levodopa doses.

The authors should include (at least as supplementary material) demographics and results of clinical assessments between PDD and DLB patients and consider repeating their analyses within each group separately if not balanced to ensure that one group is not driving their effect. This is particularly important due to the lack of significant correlation between drift rates and fluctuation scores.

REPLY: We have now included a Supplementary Table where demographics and clinical features are compared across the Parkinson's disease dementia and Dementia with Lewy bodies groups. In summary, these groups only differed significantly with respect to the Parkinson's disease dementia patients having worse motor severity scores and taking a higher daily dose of dopamine medication. Importantly, significant differences did not emerge for

cognitive or behavioural scores, and for this reason we have elected not to re-run analyses in the groups separately.

Added to the main text (page 14):

"Demographics and clinical characteristics for the Parkinson's disease dementia and dementia with Lewy bodies cases are reported separately in Supplementary material. To summarise, the groups only differed significantly with respect to Parkinson's disease dementia patients showing more severe motor features on the UPDRS-III and having a higher levodopa equivalent daily dose (See Supplementary Table 1 for details)."

Added to the Supplementary Material:

Supplementary Table 1. Mean (standard deviation) for demographics and clinical characteristics.

Demographics & clinical characteristics	Controls	AD	DLB	PDD	Group *p* value	Post hoc *p* value
**N**	23	16	18	13	-	-
**Sex (M:F)**	16:7	13:3	12:6	13:0	-	-
**Age**	76.3 (5.4)	76.4 (8.2)	77.2 (5.5)	74.3 (4.4)	n.s.	-
**Education**	11.4 (1.8)	10.6 (1.4)	10.1 (1.6)	10.9 (1.3)	n.s.	-
**MMSE**	29.1 (0.8)	22.7 (3.0)	23.9 (3.9)	24.2 (2.9)	***	AD vs. Con*** DLB vs. Con*** PPD vs. Con*** DLB vs. AD n.s. PDD vs. AD n.s. DLB vs. PDD n.s.
**CAMCOG**	96.8 (3.5)	71.6 (12.2)	76.5 (15.0)	79.5 (6.1)	***	AD vs. Con*** DLB vs. Con*** PPD vs. Con*** DLB vs. AD n.s. PDD vs. AD* DLB vs. PDD n.s.
**UPDRS-III**	1.3 (1.7)	2.0 (1.6)	14.3 (4.7)	25.6 (6.3)		AD vs. Con n.s. DLB vs. Con*** PPD vs. Con*** DLB vs. AD*** PDD vs. AD*** DLB vs. PDD***
**CAF total**	-	0.3 (0.8)	3.3 (3.9)	5.8 (3.6)	***	DLB vs. AD* PDD vs. AD*** DLB vs. PDD n.s.
**Mayo total**	-	9.0 (4.6)	11.71 (6.3)	15.5 (5.7)	*	DLB vs. AD n.s. PDD vs. AD* DLB vs. PDD n.s.

**Mayo cognitive**	-	2.1 (1.9)	2.1 (1.8)	3.5 (2.0)	n.s.	
**Mayo arousal**	-	0.9 (1.0)	1.9 (1.5)	2.9 (1.0)	***	DLB vs. AD$^{n.s.}$<br>PDD vs. AD***<br>DLB vs. PDD$^{n.s.}$
**Cholinest. Inhibitors**	-	16(100%)	16(89%)	11(85%)	-	-
**Dopaminergic meds.**		-	8 (42%)	13 (100%)	-	-
**LEDD (mg/day)**	-	-	350 (258)	889 (423)	-	**

n.s. = non significant; *** = p < .001; ** = p < .01; * = p < .05.
Significance tests refer to between group one-way ANOVAs and post hoc Sidak-corrected pairwise t-tests. AD = Alzheimer's disease; DLB = Dementia with Lewy body; PDD = Parkinson's disease dementia; MMSE = Mini-Mental State Examination; CAMCOG = Cambridge Cognitive Examination; UPDRS-III = Unified Parkinson's disease rating scale motor component; CAF total = Clinician Assessment of Fluctuation total score; Mayo total = Mayo Fluctuations Scale; Mayo cognitive = Mayo Fluctuation cognitive subscale; Mayo arousal = Mayo Fluctuations arousal subscale; LEDD = levodopa equivalent daily dose.

2. There is virtually no discussion on the study's limitations (sample size, potential MRI acquisition confounds etc), which should be incorporated in the discussion.

REPLY: We have now added a paragraph to the Discussion to explicitly raise these limitations and their effect on our study. See page 26:

"There are inherent challenges collecting fMRI task data in dementia populations. These populations are at increased risk of scanner acquisition confounds, in particular due to motion artifacts or difficulty with task execution. These limitations led to a reduced sample size in the current study, as a number of patients were excluded due to these confounds. Futures studies are needed to replicate these findings in a larger sample."

<u>Minor comments:</u>

1. Further information should be included on the imaging acquisition; was imaging acquired on or off medications for LBD participants on Levodopa, was imaging acquired at a specific time of day for all participants etc? These can have an effect on fMRI metrics (see Orban 2020, Tahmasian 2015) and should be reported.

REPLY: We had specified some of these details in the Methods already, but following the Reviewer's suggestion we have now expanded on this information.

Added to the Methods, page 7:

"Patients underwent these general assessments and the fMRI experimental task on their regular medications, with all Lewy body dementia patients in an "on" motor state, typically 1-3 hours after their last dose. **Where possible, people were scanned in the late morning to maximise attention."**

2. How many participants were excluded due to task performance and how many failed imaging QC? The authors state that the amount of excluded no-response trials did not differ between groups however no other details on excluded participant numbers are reported. This information is important to understand potential selection bias and should be included.

REPLY: We have now provided a complete breakdown of the numbers of participants excluded and for what reason. We have added these details to the Methods section.

See page 6:

"The study involved 31 people with Lewy body dementia (13 Parkinson's disease dementia and 18 dementia with Lewy bodies cases), 16 people with Alzheimer's disease, and 23 healthy controls. The participants were a subset drawn from our previous study (Firbank *et al.*, 2016, 2018). **That study originally involved 23 controls, 30 Alzheimer's disease patients, and 46 Lewy body dementia patients (22 Parkinson's disease dementia and 24 dementia with Lewy bodies cases). Four people were excluded due to technical difficulties with the response device (1 Alzheimer's disease; 3 Parkinson's disease dementia); 6 people were excluded due to scanner failures (3 Alzheimer's disease; 2 Parkinson's disease dementia; 1 dementia with Lewy bodies); 6 people were excluded after failing MRI quality checks (3 Alzheimer's disease; 3 Parkinson's disease dementia); 5 were excluded for insufficiently accurate task performance (i.e., less than 70% correct trials; 1 Parkinson's disease dementia; 4 dementia with Lewy bodies). This resulted in a cohort of 23 controls, 23 Alzheimer's disease patients, and 32 Lewy body dementia patients (13 Parkinson's disease dementia and 19 dementia with Lewy bodies cases)**, from which the participants of the current study were selected on the basis of completing a sufficient number of trials in the experimental task (further details below)."

And page 8:

"Each run of the task involved 36 trials. For the current study, we selected participants who had completed either 5 or 6 runs of the task (a total of 180 or 216 trials) and with missed responses on no more than 10% of trials**. This meant excluding 7 Alzheimer's disease patients and one dementia with Lewy bodies case from the final sample described in Firbank et al. (2016, 2018).** These measures ensured that all participants demonstrated adequate task engagement and completed a sufficient number of trials to support drift diffusion modelling."

3. Although interesting and important finding, there was no correlation between fluctuation severity and drift rates. This is surprising and limits the authors claims that impaired sensory accumulation underlies attentional deficits and fluctuations in Lewy body disease. Although mentioned in the discussion briefly, the authors should consider expanding further on this finding.

REPLY: We agree with the Reviewer that this finding deserved more attention and we have now expanded up this issue in the Discussion. See page 24:

"However, the extent of drift rate slowing did not correlate with clinical measures of fluctuation severity. This suggests that fluctuations on the trial-by-trial timescale that are

captured by the drift rate may not be well represented by clinical scales. **It may be that this reflects a broader issue with clinical scales for assessing fluctuations. These scales are a brief clinical assessment reliant on informant reports, and are therefore vulnerable to the inherent biases and inaccuracies that occur when subjective judgements are made about a symptom that is largely unobservable (Lee *et al.*, 2012; O'Dowd *et al.*, 2019; Matar *et al.*, 2020). In this sense, despite providing a useful clinical heuristic, these scales lack the granularity needed to identify meaningful variance in patients that might relate to more nuanced objective markers (Matar *et al.*, 2020). A crucial avenue for future work is to determine how various clinical and objective markers might relate to each other, and to reconcile this across the varying timescales of fluctuations.** In this respect, the hierarchical drift diffusion model offers a unique possibility to capture objective moment-by-moment fluctuations, in contrast to other neuropsychological tools that summarise performance across trials."

Editorial Office comments:

Please supply a graphical abstract. A graphical abstract is a visual representation of the central finding or methodology of the paper. It will be displayed in the table of contents. Authors should strive to make the graphical abstract informative, interesting, visually appealing, and straightforward (no caption needed, no abbreviations except gene names). Please upload as a separate high resolution image file (tiff preferred). The technical requirements for the graphical abstracts are:
--size of the submitted image: 1200 pixels square at 300dpi
--font of the text: Arial 12-16 point (smaller fonts will not be legible online)

We have included a graphical abstract with our submission.

Fig2-Fig5:for each statistical test, please report in the figure legend, the N, test used, relevant test statistic, and exact p values (for example F or t values for ANOVAs and t-tests)

These details have been added to the figure legends.
