# Supplementary material for: Impaired sensory evidence accumulation and network function in Lewy body dementia
Source: Brain Commun. 2021 Aug 2;3(3):fcab089. doi: 10.1093/braincomms/fcab089 (PMC8361397; doi:10.1093/braincomms/fcab089)
Supplement: fcab089_Supplementary_Data [file Original Submission-Decision Letter.pdf › Original Submission-Decision Letter.pdf]

Original Submission – Decision Letter

26-Feb-2021

Dear Dr. O'Callaghan,

Thank you for your recent submission BRAINCOM-2021-011
entitled "Impaired sensory evidence accumulation and network function in Lewy body dementia" to Brain Communications.

Your manuscript has been reviewed, and while the reviewers had largely positive comments, one reviewer raises some constructive critiques that I think you could address in a minor revision of the manuscript. Therefore, I invite you to respond to the reviewers' comments and revise your manuscript. In particular, we will ask you to address the two main comments raised by the second reviewer.

The reviewers' comments are included at the bottom of this message.

In addition to the reviewers' comments, please note our editorial comments added below to comply with our requirements for presentation of scientific data at Brain Communications.

Please format your revised manuscript according to our Instructions to Authors https://academic.oup.com/braincomms/pages/General_Instructions – a summary of key points is attached to help with this.

To revise your manuscript, click the link below or log into https://mc.manuscriptcentral.com/braincom and enter your Author Centre, where you will find your manuscript title listed under "Manuscripts with Decisions." Under "Actions," click on "Create a Revision." Your manuscript number has been appended to denote a revision.

*** PLEASE NOTE: This is a two-step process. After clicking on the link, you will be directed to a webpage to confirm. ***

https://mc.manuscriptcentral.com/braincom?URL_MASK=e9acd7834ca544ad8718fc65d25cdd63

Please upload a revised manuscript with the changes you have made shown either with "track changes" or in coloured text.

When submitting your revised manuscript, you will be asked to respond to the reviewer(s) comments. Please be as specific as possible in your response to all of the comments.

The editorial team at Brain Communications also aims to increase transparency in scientific publishing and to demonstrate the value that peer review brings to the scientific process. Please therefore consider whether you are willing to have reviewer comments on your paper and your response published as supplementary data with the paper if it is accepted. This is entirely optional and will only be done if both the authors and reviewers agree. When submitting your revision you will have the opportunity to update the answer you entered previously.

IMPORTANT: Your original files are available to you when you upload your revised manuscript. Please delete any redundant files before completing the submission.

Because we are trying to facilitate timely publication of manuscripts submitted to Brain Communications, we would appreciate your revised manuscript as soon as possible. If it is not possible for you to resubmit within 3 months, the option to submit a revision will expire, and we would then consider your revised paper as a new submission. However, if you need a little more time, please get in touch and we can extend this deadline for you.

Once again, thank you for submitting your manuscript to Brain Communications and I look forward to receiving your revision.

Yours sincerely,
Dr. Alberto Lleo
Editor, Brain Communications

Reviewer(s)' Comments to Author:

Reviewer: 1 ( Klaus Ebmeier )

Comments to the Author
Interesting and competent work, well presented.
Typos:
Imaging acquisition -> Image acquisition?

Reviewer: 2 (Angeliki Zarkali )

Comments to the Author
The authors use a modified attentional task and computational modelling to assess sensory evidence accumulation in patients with Lewy body disease compared to Alzheimer's patients and controls. The attentional task and drift diffusion modelling are well described and well designed to test the authors hypothesis. The authors find impaired evidence accumulation in LBD but no correlation between drift rates and measures of fluctuation or cognitive measures. The findings are interesting and the additional correlation with resting state functional networks provides further useful insights into the cognitive mechanisms of LBD. However, there are few areas that further methodological information is needed.

Major comments
1. The authors rightly include both patients with Parkinson's disease dementia and Dementia with Lewy bodies in their LBD patient group; although underlying pathophysiology is likely to be similar in both groups they may differ in key factors, particularly in the degree of cognitive impairment, the presence and severity of fluctuations and levodopa doses.
The authors should include (at least as supplementary material) demographics and results of clinical assessments between PDD and DLB patients and consider repeating their analyses within each group separately if not balanced to ensure that one group is not driving their effect.
This is particularly important due to the lack of significant correlation between drift rates and fluctuation scores.
2. There is virtually no discussion on the study's limitations (sample size, potential MRI acquisition confounds etc), which should be incorporated in the discussion.

Minor comments:
1. Further information should be included on the imaging acquisition; was imaging acquired on or off medications for LBD participants on Levodopa, was imaging acquired at a specific time of day for all participants etc? These can have an effect on fMRI metrics (see Orban 2020, Tahmasian 2015) and should be reported.
2. How many participants were excluded due to task performance and how many failed imaging QC? The authors state that the amount of excluded no-response trials did not differ between groups however no other details on excluded participant numbers are reported. This information is important to understand potential selection bias and should be included.
3. Although interesting and important finding, there was no correlation between fluctuation severity and drift rates. This is surprising and limits the authors claims that impaired sensory accumulation underlies attentional deficits and fluctuations in Lewy body disease. Although mentioned in the discussion briefly, the authors should consider expanding further on this finding.

Editorial Office comments:

-Please supply a graphical abstract. A graphical abstract is a visual representation of the central finding or methodology of the paper. It will be displayed in the table of contents. Authors should strive to make the graphical abstract informative, interesting, visually appealing, and straightforward (no caption needed, no abbreviations except gene names). Please upload as a separate high resolution image file (tiff preferred). The technical requirements for the graphical abstracts are:
--size of the submitted image: 1200 pixels square at 300dpi
--font of the text: Arial 12-16 point (smaller fonts will not be legible online)

-Fig2-Fig5:for each statistical test, please report in the figure legend, the N, test used, relevant test statistic, and exact p values (for example F or t values for ANOVAs and t-tests)
